# The phospholipase PlcP mediates membrane lipid remodeling required for the intracellular survival of *Burkholderia cenocepacia*

**DOI:** 10.1016/j.jbc.2026.113600

**Published:** 2026-09-23

**Authors:** Holly Shropshire, Xu Han, Zengsheng Han, Barbara Clough, Rebekah A. Jones, Shadman Ahmed, Inmaculada García-Romero, Isabel Aberdeen, Prachetash Ghosh, Ioannis Nezis, Miguel A. Valvano, David J. Scanlan, Richard Guillonneau, Yin Chen

**Affiliations:** 1 School of Life Sciences, University of Warwick, Coventry, UK; 2 State Key Laboratory of Metastable Materials Science and Technology, Nano-biotechnology Key Lab of Hebei Province, Applying Chemistry Key Lab of Hebei Province, Yanshan University, Qinhuangdao, China; 3 School of Biosciences, University of Birmingham, Birmingham, UK; 4 Wellcome-Wolfson Institute for Experimental Medicine, Queen’s University Belfast, Belfast, UK; 5 Nantes Université, CNRS, US2B, UMR6286, Nantes, France; 6 Institute of Microbiology and Infection, University of Birmingham, Birmingham, UK

**Keywords:** lipid remodelling, plcP, Burkholderia cenocepacia, cystic fibrosis pathogens, intracellular pathogens

## Abstract

*Burkholderia cenocepacia* is a problematic pathogen that infects people with cystic fibrosis and can cause fatal "cepacia syndrome". *B. cenocepacia* infection is difficult to treat due to the high-level intrinsic resistance of the bacterium to antimicrobials and its ability to survive in macrophages. In this study, we uncover a hitherto unknown aspect of *B. cenocepacia*’s pathogenesis related to the formation of new glyceroglycolipids, which are involved in intracellular survival. Using lipidomics, we observed that *B. cenocepacia* can produce three glyceroglycolipid species in phosphate deplete conditions using a PlcP-mediated lipid remodeling pathway originally discovered in soil and ocean-dwelling bacteria. While lipid remodeling as an adaptive strategy for environmental microbes to overcome phosphorus limitation is known, its role in intracellular bacterial survival has not been investigated. Using mammalian macrophages and *Galleria mellonella* larvae as infection models, we show that mutants unable to perform membrane lipid remodeling (Δ*plcP*, Δ*agt*) could not establish an infection. Unlike the wild-type bacterium, the Δ*plcP* mutant does not replicate within macrophages. Comparative genomics analyses showed that this PlcP-Agt pathway is conserved in all pathogenic *Burkholderia* that infect mammalian and plant hosts. Overall, our results indicate that bacterial membrane lipid remodeling plays an essential role in the intracellular survival of *B. cenocepacia*.

*Burkholderia cenocepacia* belongs to the *Burkholderia cepacia* complex (Bcc), a group of at least 20 closely related *Burkholderia* species (1). These are opportunistic human pathogens that typically infect people with cystic fibrosis and chronic granulomatous disease (CGD) (2, 3). The most prevalent bacterial species infecting cystic fibrosis patients is *Pseudomonas aeruginosa*, which has been extensively studied (4, 5). However, Bcc infections are problematic due to the high-level intrinsic resistance of *Burkholderia* species to antimicrobials (6, 7), and the transmissibility of the infection across patients (8, 9); thus, such infections are a contraindication to lung transplantation (10, 11).

*B. cenocepacia* and other *Burkholderia* species can survive in immune cells such as macrophages (12, 13), a common strategy for many intracellular bacterial pathogens (14). Infected hosts have many countermeasures to restrict pathogens, including withholding essential nutrients for microbial growth, a process known as nutritional immunity. This is best exemplified by the restriction of transition metals that are essential for microbial metabolism (15). Recent evidence suggests that nutritional immunity can be extended to other essential microbial nutrients such as phosphorus (16, 17). Microbial pathogens have evolved sophisticated mechanisms to combat host restriction of essential nutrients. For example, *Salmonella* can adapt to phosphorus depletion during infection by upregulating the phosphorus starvation response, including upregulation of high-affinity phosphate (Pi) uptake systems and utilization of host-derived organic phosphorus to overcome phosphorus limitation during host infection (14, 17).

Membrane lipid remodeling is another bacterial strategy to cope with phosphorus limitation, which is widely studied in environmental microbes (18, 19, 20, 21) but not systematically investigated in human pathogens. Bacterial membranes typically contain glycerophospholipids such as phosphatidylglycerol (PG) and phosphatidylethanolamine (PE). However, bacterial membrane lipids are much more complex and dynamic than previously thought (22). We and others have shown that several microbes can substitute phosphorus-containing glycerophospholipids with surrogate non-phosphorus lipids to save the cellular phosphorus quota (18, 19, 20, 21, 22, 23, 24, 25). This process requires a phospholipase C-type enzyme, PlcP, to produce phosphorus-free membrane lipids. For example, the PlcP-mediated lipid remodeling pathway allows cosmopolitan marine heterotrophic bacteria to be as competitive as their photosynthetic counterparts and to thrive in world’s most oligotrophic marine surface waters (19, 26). In the legume-associated symbiotic bacterium, *Sinorhizobium meliloti*, a mutant unable to perform lipid remodeling grows poorly in phosphate limiting conditions, becoming less competitive in the natural environment (18). The PlcP pathway is also present in several major bacterial pathogens; we previously showed in *P*. *aeruginosa* that PlcP plays a role in lipid remodeling that affects antimicrobial resistance (21). However, how lipid remodeling modulates infection is unknown. In this study, we investigated the link between lipid remodeling and virulence using the intracellular pathogen *B. cenocepacia*. We reveal that lipid remodeling is required for *B. cenocepacia* virulence and contributes to the bacterium's intracellular survival.

## Results

### *B. cenocepacia* K56-2 produces glycolipids under phosphorus limitation

We have previously shown that *B. cenocepacia* K56-2 produces inorganic phosphate (Pi) scavenging proteins when grown under Pi-limited conditions (27). Re-analysis of the previously published proteomics dataset identified three proteins encoded by a putative 3-gene operon, which were significantly upregulated under Pi-depletion (27). This operon encoded a predicted PlcP phospholipase (BCAL2364), a putative glycosyltransferase involved in glycoglycerolipid synthesis (BCAL2365, *agt*), and a putative diacylglycerol kinase (BCAL2366, *dagK*) (Fig. 1*A*). We hypothesized that this operon was involved in membrane lipid remodeling in response to Pi-deficiency in *B*. *cenocepacia* (Fig. 1*A*). In this model PlcP produces the diacylglycerol intermediates needed for glycolipid synthesis *via* Agt, while the putative diacylglycerol kinase recycles excess diacylglycerol back into phospholipid synthesis to prevent its accumulation in the membrane (28).

**Figure 1:**
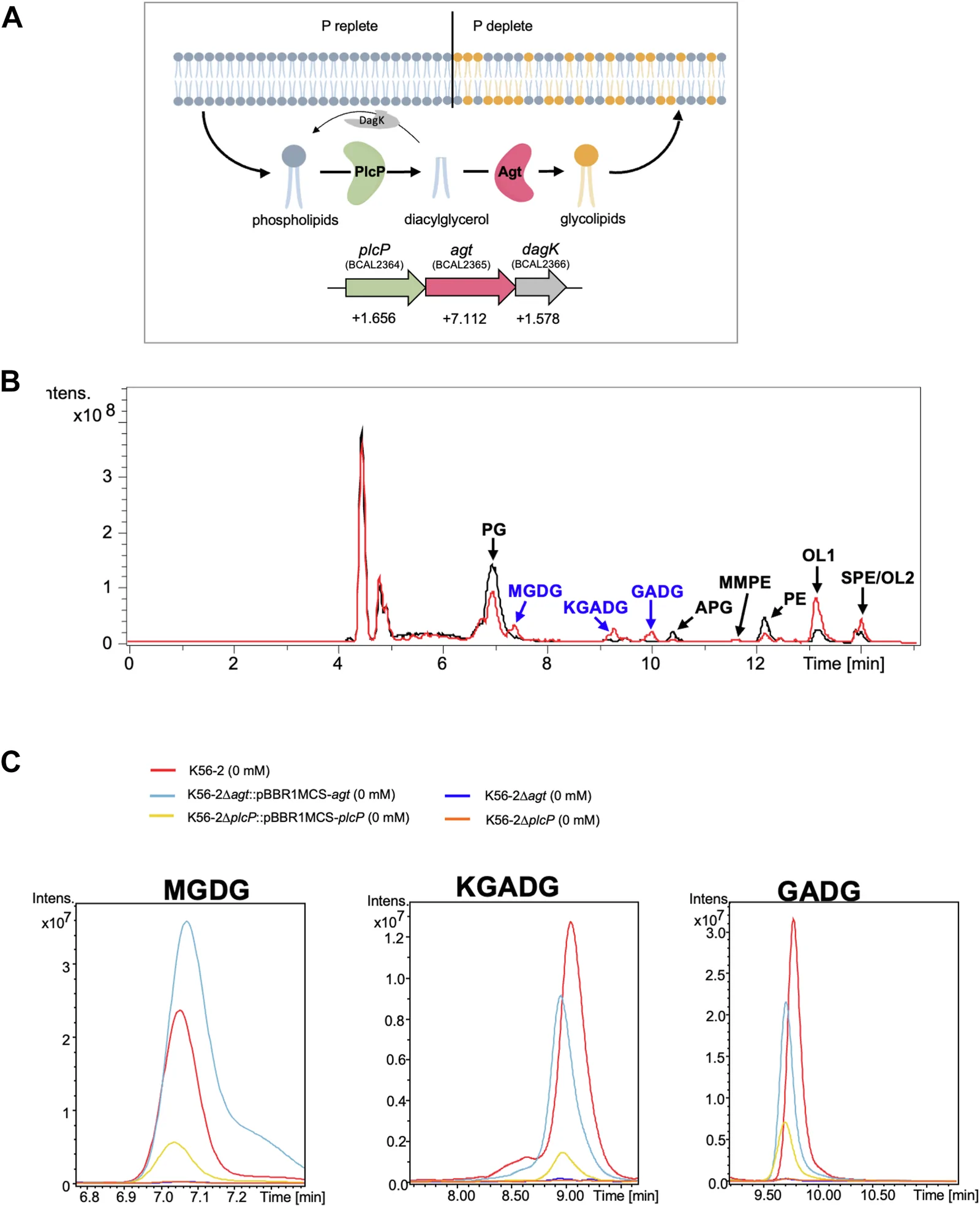
***B. cenocepacia* remodels its membrane lipids under phosphorus limitation.***A*, the working model of key enzymes involved in lipid remodeling in *B. cenocepacia*. The lipid remodeling operon in *B. cenocepacia* consists of *plcP* (BCAL2364), *agt* (BCAL2365) and *dagK* (BCAL2366). Below the operon are the log_2_(fold change) values of these proteins in Pi-depletion compared to Pi-replete conditions identified in our previous comparative proteomics dataset (27), showing that these proteins are significantly upregulated under Pi-limitation. *B*, full chromatogram, in positive ionization mode, of the *B. cenocepacia* lipidome under Pi-deplete (*red*) and Pi-replete (*black*) growth conditions. Lipids are indicated as follows: APG, alanyl-phosphatidylglycerol; GADG, glucuronic acid diacylglycerol; KGADG, keto-glucuronic acid diacylglycerol; MGDG, monoglucosyl diacylglycerol; MMPE, monomethylated phosphatidylethanolamine; OL1, OL2, ornithine lipid type 1 and 2; PE, phosphatidylethanolamine; PG, phosphatidylglycerol; SPE, sphingosyl-phosphatidylethanolamine (internal standard). *C*, extracted ion chromatograms of the glycolipids MGDG, KGADG and GADG in positive ionization mode (*m/z* 760.6, 772.6, and 814.6, respectively), which were extracted after 24 h of growth in MMA. *Red* chromatogram, wild type under Pi-depletion; *dark orange* and *dark blue* chromatograms, Δ*plcP* and Δ*agt* mutants, respectively, under Pi-depletion; *yellow* and *light blue* chromatograms, complementation strains (K56-2Δ*plcP*::pBBR1MCS-*plcP*, K56-2Δ*agt*::pBBR1MCS-*agt*) under Pi-depletion.

We investigated the membrane lipid profile of *B. cenocepacia* K56-2 when grown under Pi-limitation by comparative lipidomics *via* liquid chromatography-mass spectrometry (LC-MS) at the end of an 8-h growth period in Pi-deplete (0 mM) and Pi-replete (1 mM) defined minimal medium (modified minimal A medium, MMA) (21) (Fig. 1*B*). LC-MS analysis identified three lipids that were present in the parental K56-2 under Pi-depletion (Fig. 1*C*) but absent in the Pi-replete condition (Fig. 1*B*). MS^n^ fragmentation of the lipids eluting at ∼7.1 min, ∼9.1 min and ∼9.7 min showed a neutral loss of 179, 191 and 193, respectively (Fig. 2*A*). Such a neutral loss and MS^n^ fragmentation pattern is consistent with glycolipids with a glucosyl (neutral loss of 179) and glucuronosyl (neutral loss of 193) hydrophilic headgroups, suggesting the glycoglycerolipids eluted at 7.1 min and 9.7 min are monoglucosyl diacyglycerol (MGDG) and glucuronosyl diacylglycerol (GADG), respectively. MGDG and GADG lipids are produced by several bacteria in response to Pi limitation and the hydrophilic hexose and hexanoate head groups have been characterized previously using nuclear magnetic resonance (19, 20, 21). The neutral loss of 191 from the lipid eluted at 9.1 min suggests that the hydrophilic head group is likely a dehydrogenated hexonate. Such a keto-hexonate has been observed previously in the synthesis of UDP-xylose and UDP-D-galacturonate from UDP-glucuronate (29, 30). Thus, we tentatively assign this lipid as ketoglucuronosyl diacylglycerol (KGADG). The production of these glycolipids required both PlcP and Agt since they were absent in K56-2Δ*plcP* and K56-2Δ*agt* mutants but detected in their corresponding complemented strains Δ*plcP*::pBBR1MCS-*plcP* and Δ*agt*::pBBR1MCS-*agt* (Fig. 1*C*).

**Figure 2:**
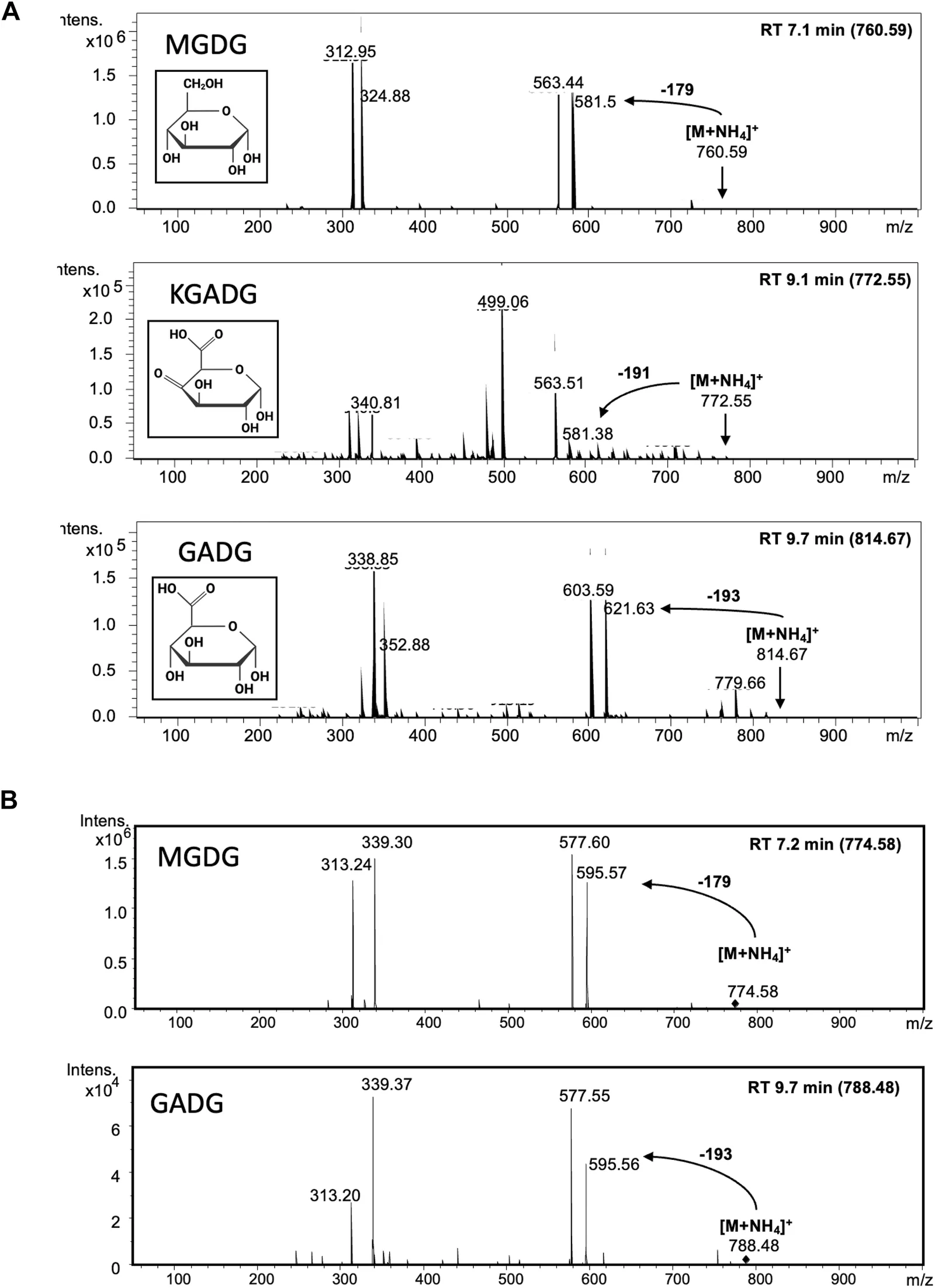
**Characterization of glycolipids by mass spectrometry.***A*, MS (2) fragmentation of glycolipids, MGDG, GADG and KGADG. The MGDG peak at 7.1 min with parent ion 760.59, showing a neutral loss of 179 *m/z* corresponding to the loss of the glucosyl head group. The KGADG peak at 9.1 min with parent ion 772.55, showing a neutral loss of 191 m/z corresponding to the loss of the keto-glucuronosyl head group. The GADG peak at 9.7 min with parent ion 814.67, showing a neutral loss of 193 *m/z* corresponding to the loss of the glucuronosyl head group. All fragmentations are shown in positive ionization mode. *B*, MS (2) fragmentation of MGDG and GADG species obtained from recombinant *Escherichia coli* harbouring the putative glycosyltransferase *agt* of *Burkholderia cenocepacia* K56-2. The two peaks corresponding to monoacylglycerol with 18:1 (m/z 339) and 16:0 (m/z 313) fatty acid, respectively.

To further investigate the role of the putative glycosyltransferase BCAL2365 (*agt*) in glycolipid production, we cloned and overexpressed the *agt* gene in recombinant *Escherichia coli*. As shown in Figure 2*B*, overexpressing *agt* was sufficient to enable *E. coli* to produce MGDG and GADG, though the putative KGADG was not detected; as expected, no glycolipids were observed in the empty plasmid control (Fig. S1). Together, these experiments indicate that *B. cenocepacia* can remodel its membrane in response to Pi limitation by substituting membrane phospholipids with non-phosphorus containing glycoglycerolipids.

### PlcP-mediated lipid remodeling is required for pathogenicity in the *Galleria mellonella* infection model

*B. cenocepacia* is a facultative intracellular pathogen that survives in membrane vacuoles (13). Since Pi can be a limiting nutrient for survival and replication of bacterial pathogens in host cells (17, 31), we investigated whether Pi stress-induced lipid remodeling plays a role in the intracellular lifestyle of *B. cenocepacia*. We thus investigated whether PlcP- and Agt-mediated lipid remodeling is required for *B. cenocepacia* virulence *in vivo* using the *G. mellonella* larvae infection model. Survival of infected larvae was assessed over a 48-h period post-infection after injection with wild-type K56-2, Δ*plcP*, Δ*agt* and their corresponding complemented strains. In addition to live/dead assay by counting the live/dead larvae post-infection, we also adopted a standardized combined health index scoring system, which also considers the progressive myelinization of larvae, cocoon formation, and movement in addition to survival (32). The percentage of the wild-type infected surviving larvae decreased rapidly between 24 and 48 h post-infection, and all larvae were dead by 48 h (Fig. 3*A*) and a one-way ANOVA test showed a significant effect of the *plcP/agt* mutation (*p* < 0.001) on larval survival. Infection with either the *plcP* mutant or the *agt* mutant strains caused significantly fewer deaths (76 ± 6%, 70 ± 10% survived respectively; *p* < 0.001 *post hoc* Dunnett’s test). This suggests that the PlcP/Agt-mediated lipid remodeling pathway is important for the bacterium to establish infection in the insect. This is further supported by the combined health score assay since the comparison of wild-type-infected larvae with those infected by the mutants showed that the health index of the larvae infected by the wild-type strain decreased rapidly over 48 h (Fig. S2). In contrast, the health index of the Δ*plcP-* and Δ*agt-*infected larvae only decreased by 29 ± 9% and 34 ± 3%, respectively, at 48 h, confirming that loss of *plcP/agt* causes a significant defect for the bacterium to establish an infection in *G. mellonella*. A representative photograph of this experiment can be seen in Figure 3*B*, highlighting the clear difference in survival of infected larvae after 48 h. Control experiments using larvae infected with heat-inactivated wild type showed that all larvae survived across the 48-h period (Fig. 3*A*). The virulence of the Δ*plcP/*Δ*agt* mutants in the *G. mellonella* infection model was partially rescued by expressing *plcP* or *agt* from their native promoters from a broad-host-range vector for Gram-negative bacteria. We attributed this partial complementation to plasmid instability in the absence of antibiotic selection, which led to partial plasmid loss during infection in *G. mellonella* (Fig. S3).

**Figure 3:**
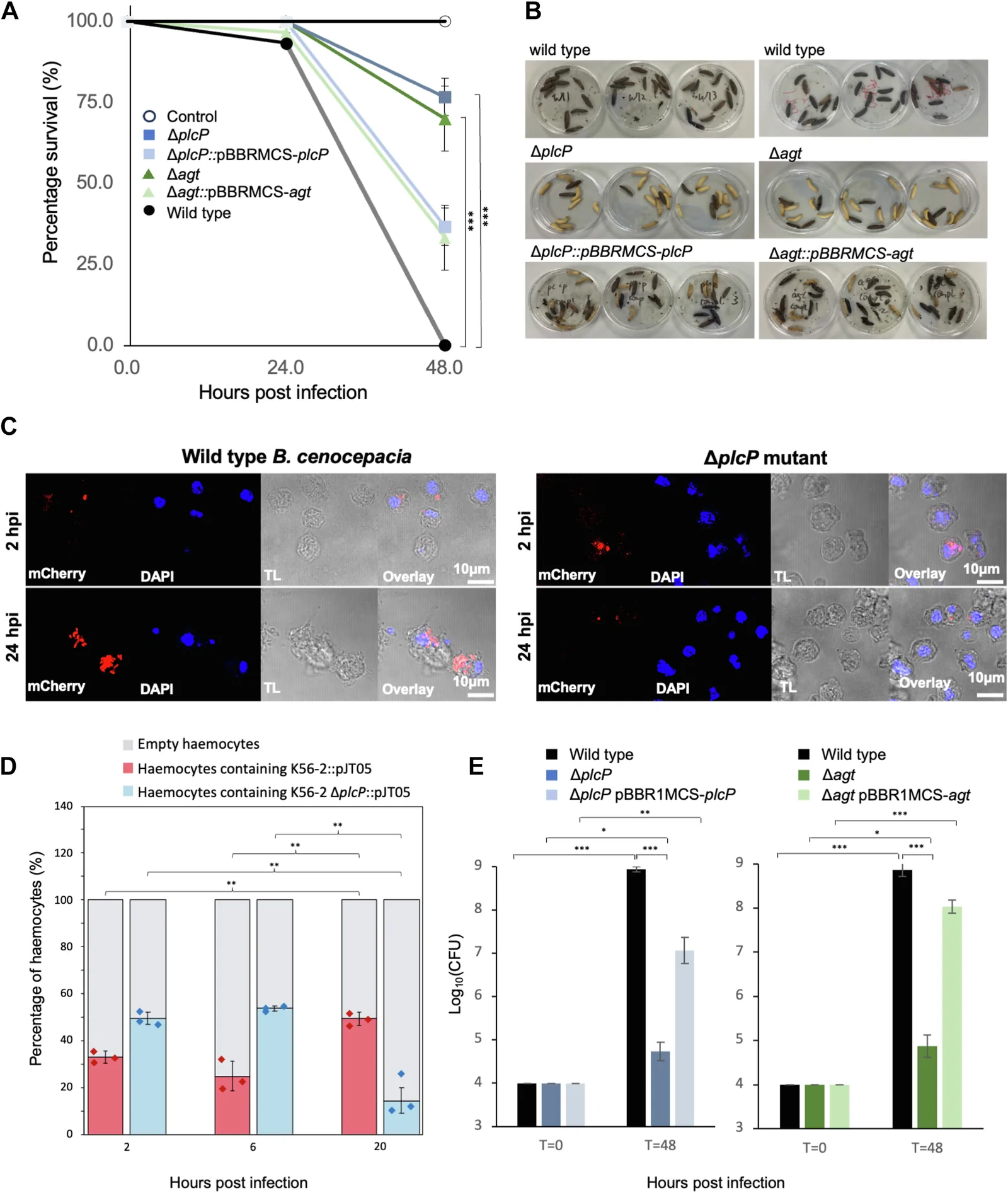
**The *plcP/agt* mutants of *B. cenocepacia* are unable to establish successful infection in the *Galleria mellonella* infection model.***A*, *G. mellonella* larvae survival when infected with the wild type *B. cenocepacia* K56-2 (*black circles*), Δ*plcP* mutants (*dark blue squares*) and the Δ*plcP* complemented mutant (*light blue squares*) and Δ*agt* mutants (*dark green triangles*) and the Δ*agt* complemented mutant (*light green triangles*). Heat-inactivated K56-2 (*white circles*) controls were also included, and all larvae survived during the 48 h experiment. Error bars represent ± standard deviation (3 biological replicates, 10 larvae per replicate). ∗∗∗, *p* < 0.001, *post hoc* Dunnett's test. *B*, photograph of the experiment in Figure 3A showing the three biological replicates at 48 hpi, highlighting the stark contrast in melanization of *G. mellonella* larvae between wild-type K56-2 and the Δ*plcP/agt* mutants. *C*, confocal microscopy of the wild type and the *plcP* mutant containing the mCherry-expressing plasmid pJT05 in haemocytes at 2- and 24-hpi. *Red* fluorescence indicates mCherry-expressing bacterial cells; *blue* fluorescence indicates DAPI staining of the hemocyte nucleus. TL: transmitted light microscopy image. Scale bars indicate 10 μm. Wild-type bacteria replicate intracellularly after 24 h contrary to the mutant which did not replicate intracellularly. *D*, the percentage of haemocytes that contain at least 1 bacterial cell of K56-2 (*red*) and K56-2Δ*plcP* (*blue*). ∼100 cells were counted in triplicate. Error bars represent ± standard deviation. ∗∗*p* ≤ 0.01, independent *t* test. *E*, enumeration of wild-type (*black*), Δ*plcP* mutant (*blue*) and Δ*agt* mutant (*green*) from infected *G. mellonella* larvae at 0 and 48 hpi shown as log_10_ CFU/ml. Error bars represent ± standard deviation. ∗, *p* < 0.05; ∗∗∗, *p* < 0.001, independent *t* test.

To investigate whether bacterial survival was compromised during the infection inside of the insect, larvae were culled, and insect hemolymph was visualized under confocal microscopy. Since both the Δ*plcP* mutant and Δ*agt* mutant exhibited similar lipid profiling phenotypes and reduced virulence in *G. mellonella* infection assays, we used the Δ*plcP* mutant for the subsequent experiments. At 24 h post-infection (hpi) wild type, but not K56-2Δ*plcP* bacteria, were present in hemocytes (Fig. 3*C*), as determined by the percentage of hemocytes containing bacterial cells (Fig. 3*D*). This is also supported by transmission electron microscopy (TEM), which showed the wild type bacteria present inside *Burkholderia*-containing vacuoles within infected haemocytes (Fig. S4). Further, between 0 and 48 h post-infection (hpi), the numbers of wild type bacteria compared with Δ*plcP* or Δ*agt* mutants increased significantly in the extracted haemolymph (Fig. 3*E*), suggesting that the mutants are unable to survive or replicate in the larvae.

### *plcP* is actively expressed in hemocytes and the mutant is more likely to associate with acidified vacuoles

We also investigated the timing of *plcP* expression during *Galleria* infection. For these experiments, we constructed a fluorescence-based reporter by fusing the *plcP* promoter region with a gene encoding mCherry, allowing us to track *plcP* expression in *Galleria* haemocytes. mCherry expression became detectable at 30 min post-infection (Fig. 4*A*), suggesting early activity of the *plcP* promoter following bacterial engulfment. At 1 hpi, ∼60% of the bacteria expressed mCherry.

**Figure 4:**
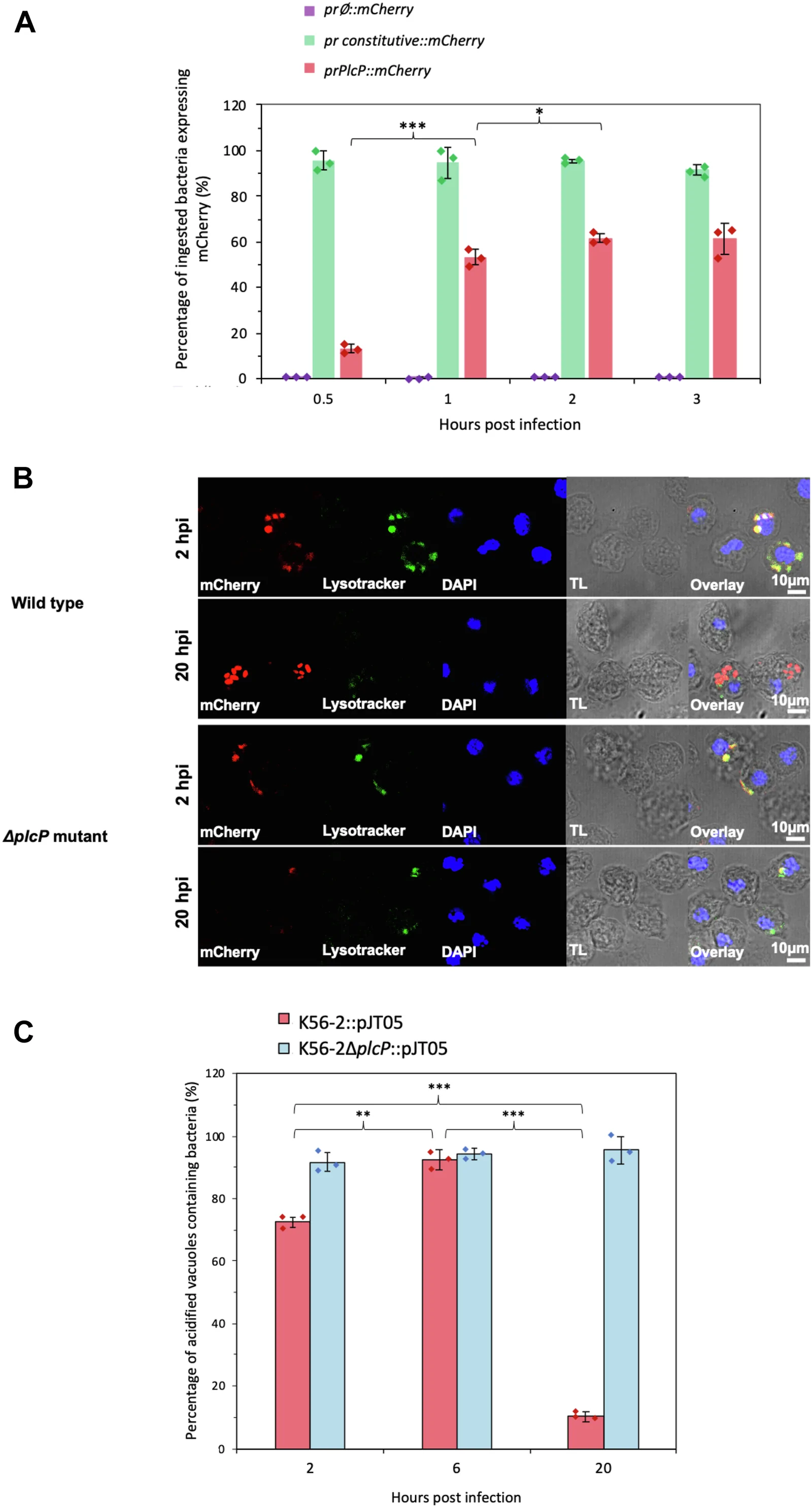
**PlcP expression and phagolysosome acidification in *G. mellonella* haemocytes during infection.***A*, expression of PlcP in haemocytes using K56-2 with plasmids expressing mCherry from the putative *plcP* promoter (*prPlcP*::*mCherry*), expressing mCherry constitutively (*pr* constitutive*::mCherry*) and mCherry with no promoter (*pr∅::mCherry*). ∼100 cells were counted in triplicate. Error bars represent ± standard deviation. ∗*p* ≤ 0.05, ∗∗∗*p* ≤ 0.001, independent *t* test. *B*, confocal microscopy of K56-2 and K56-2Δ*plcP* containing mCherry-expressing plasmid pJT05 in haemocytes at 2- and 20-hpi. *Red* fluorescence indicates mCherry-expressing bacterial cells, *green* fluorescence indicates acidification with Lysotracker *green*, *blue* fluorescence indicates DAPI staining of the hemocyte nucleus. TL: transmitted light microscopy image. Scale bars indicate 10 μm. *C*, percentage of haemocytes containing K56-2 (*red*) and K56-2Δ*plcP* (*blue*) localized within an acidified compartment. ∼100 cells were counted in triplicate. Error bars represent ± standard deviation. ∗∗∗*p* ≤ 0.001, independent *t* test.

Intramacrophage survival of *B. cenocepacia* occurs in a late phagosomal compartment with delayed acidification (33). Therefore, we investigated whether PlcP expression is associated with phagosome acidification using confocal microscopy of *G. mellonella* haemocytes infected with mCherry-labeled bacteria and stained with Lysotracker Green. At 2 hpi, both wild-type and the Δ*plcP* mutant bacteria were associated with acidified vacuoles (Fig. 4*B*). By 20 hpi, wild-type bacteria showed significant intracellular growth within haemocytes (Fig. 4*B*) and were no longer associated with the Lysotracker marker (Fig. 4*C*). In contrast, very few Δ*plcP* mutant bacteria were detected in hemocytes at 20 hpi (Fig. 4*B*), but most of those observed remained associated with the Lysotracker marker (Fig. 4*C*). Therefore, we conclude that, unlike the wild-type strain that can modify the acidic endosome (34, 35, 36), the Δ*plcP* mutant fails to replicate intracellularly, becoming nearly cleared from the hemocytes (Figs. 3*E* and 4*C*).

### PlcP is also required for intracellular survival of *B. cenocepacia* in macrophages

To uncover whether lipid remodeling is also required for infection in macrophages, a *plcP* deletion mutant (Δ*plcP*) was generated in strain MH1K. This strain is a gentamicin-sensitive derivative of *B. cenocepacia* K56-2, which enables the enumeration of the intracellular bacterial load using the gentamicin protection assay (37). Murine macrophage RAW264.7 were cultured in a Pi-limited medium during the periods of interaction with the bacteria. At 2 hpi, internalized WT and Δ*plcP* were observed by confocal microscopy at comparable numbers, but at 24 hpi, the number of Δ*plcP* bacteria within infected macrophages was reduced compared with WT bacteria-infected cells (Fig. 5*A*). Quantitative analysis by calculating the percentage of infected macrophages containing at least one bacterial cell shows the percentage of macrophages containing *B. cenocepacia* WT significantly increased over 24 h of infection and nearly all macrophages were infected by the bacterium at 24 hpi. In contrast, the percentage of macrophages infected with the *plcP* mutant did not change significantly over time (Fig. 5*B*). This suggests that Δ*plcP* has a defect in intracellular survival, which was further supported by the analyses of intracellular bacterial loads (colony forming units, CFU) at each timepoint post-infection (Fig. 5*C*). From 6 hpi to 24 hpi, there was a significant increase in bacterial cell numbers (∼2 log units) for the wild type, as previously described (29), whereas the mutant cell numbers were significantly lower (*p* ≤ 0.05, *t* test) (Fig. 5*C*). Therefore, the lack of replication of Δ*plcP* in macrophages suggests that lipid remodeling is essential to overcome macrophage immunity, ensuring successful intracellular replication and long-term survival of *B. cenocepacia*.

**Figure 5:**
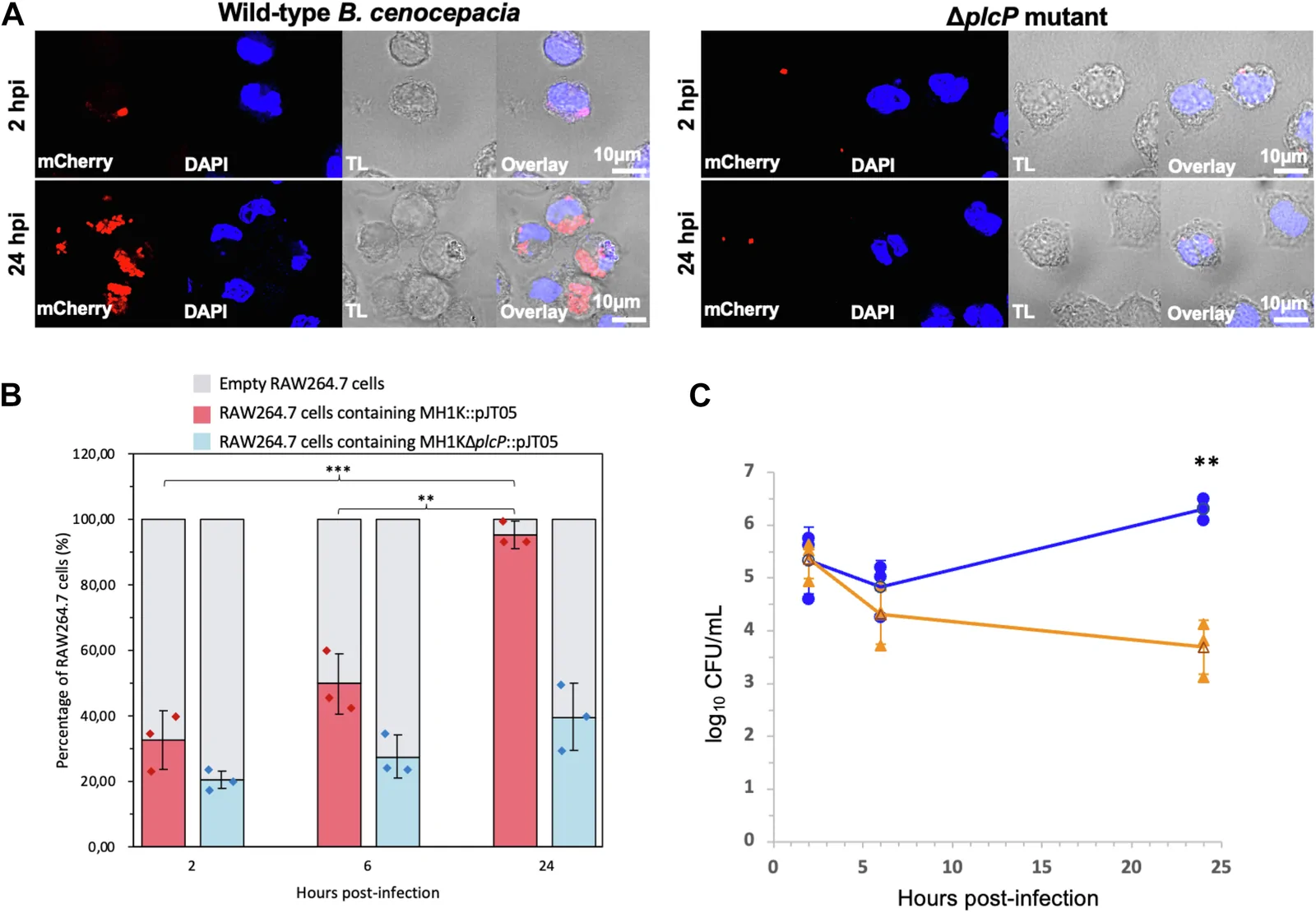
**The *plcP* mutant of *B. cenocepacia* shows a defect in intracellular survival in RAW264.7 macrophages.***A*, confocal microscopy of the wild type (MH1K) and the *plcP* mutant (MH1KΔ*plcP*) containing mCherry expressing plasmid pJT05 in RAW264.7 macrophages at 2 and 24 hpi. *Red* fluorescence indicates mCherry expressing bacterial cells, and *blue* fluorescence indicates DAPI staining of the macrophage nucleus. TL: transmitted light microscopy image. Scale bars indicate 10 μm. Wild type bacteria replicate after 24 h intracellularly contrary to the Δ*plcP* mutant which did not replicate intracellularly. *B*, the percentage of RAW264.7 macrophages which contain at least 1 bacterial cell of the wild type (*red*) or the mutant (*blue*) expressing the mCherry plasmid, pJT05. Around 100 cells were counted in triplicate and error bars represent ± standard deviation. A significant increase in the wild type containing macrophages can be seen over time, but not for the mutant containing macrophages. ∗∗*p* ≤ 0.01, ∗∗∗*p* ≤ 0.001 (Independent *t* test). *C*, bacterial survival measured as log_10_ CFU/ml recovered from RAW264.7 infected macrophages. Significant difference in CFU/ml between the wild type and the mutant at 24 hpi (∗∗, *p* ≤ 0.01, independent *t* test). Error bars represent ± standard deviation (n = 3).

### PlcP-mediated lipid remodeling pathway is conserved in pathogenic *Burkholderia* spp

To examine the conservation of the *plcP* pathway in the *Burkholderia* genus, we searched the genome-sequenced *Burkholderia* strains (1302 genomes) deposited in the Integrated Microbial Genome and Microbiomes database at the Joint Genome Institute (JGI). The genus *Burkholderia* was first proposed in 1992 and later split into *Burkholderia* spp. (pathogenic strains) and *Paraburkholderia* spp. (non-pathogenic environmental isolates) based on comprehensive phylogenetics analyses (38, 39). The phylogeny of *Burkholderia* spp. was reconstructed using the housekeeping *rpoB* gene, resulting in a phylogenetic tree that classifies pathogenic *Burkholderia* into three distinct clades (Fig. 6*A*). Clade Ia includes the Bcc strains, such as *B. cenocepacia*, *B. cepacia* and *Burkholderia multivorans.* Clade Ib includes *Burkholderia pseudomallei* and *Burkholderia mallei*, causative agents of human melioidosis and mammalian glanders, respectively, whereas Clade Ic includes several phytopathogens (*e.g.*, *Burkholderia glumae*, *Burkholderia gladioli* and *Burkholderia plantarii*) (39). All the genomes from species grouped into these three clades carried a predicted *plcP-agt-dagK* operon with a PlcP orthologue showing >84%, 91%, 72% amino acid sequence identity to the K56-2 PlcP, respectively (Fig. 6*B*). Thus, comparative genomics suggests that the ability to perform lipid remodeling through the PlcP pathway is conserved in pathogenic *Burkholderia* spp.

**Figure 6:**
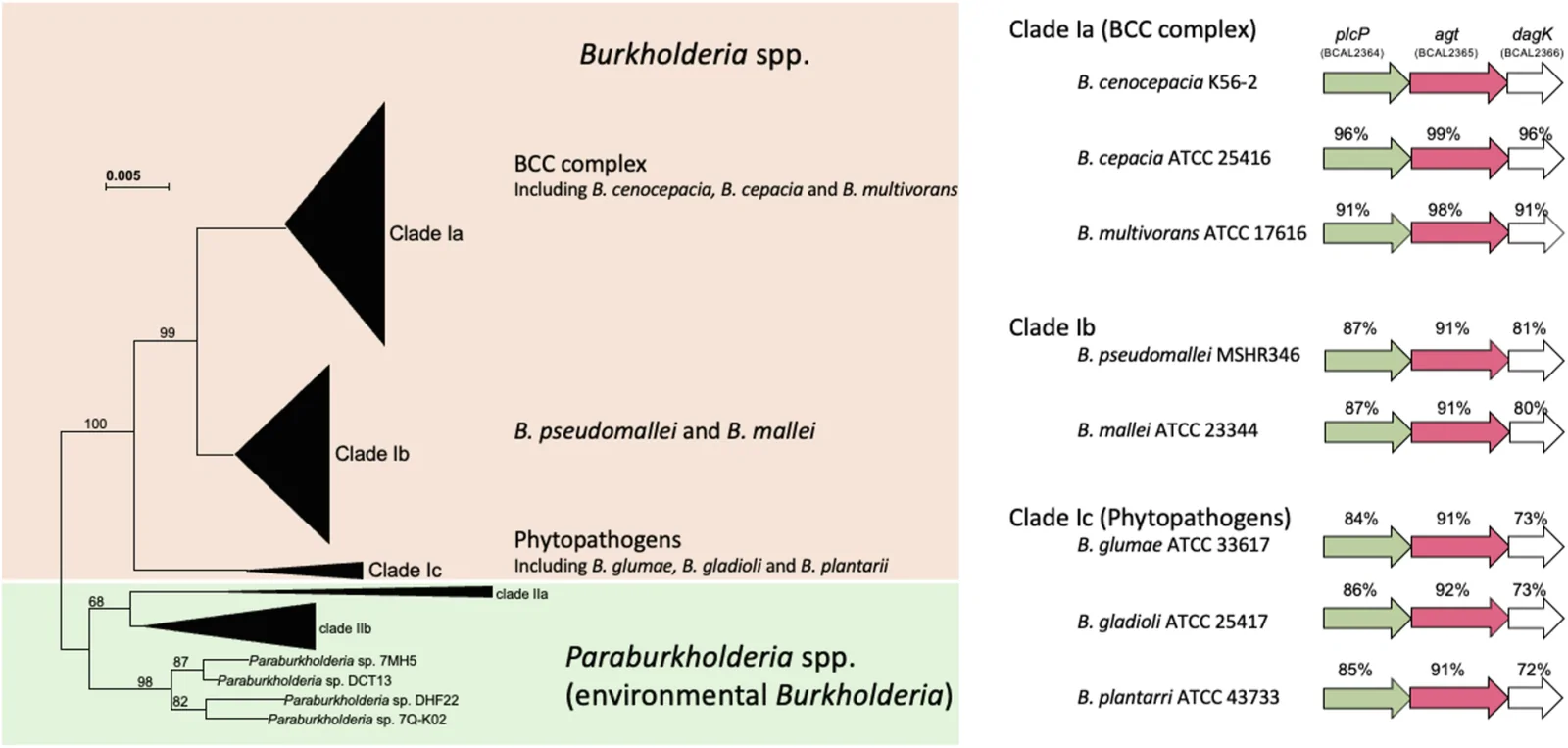
**Comparative genomic analysis of the distribution of the PlcP pathway in *Burkholderia* species, including human, animal and plant pathogens.***Left*, the evolutionary history of the housekeeping RpoB protein was inferred using the Neighbor-Joining method. RpoB from the newly established *Paraburkholderia* spp. (environmental *Burkholderia* isolates) was used as the outgroup. Clade Ia includes all Bcc complex bacteria; Clade Ib includes pathogenic *B. pseudomallei* and *B. mallei*; and Clade Ic includes phytopathogenic strains. The percentage of replicate trees in which the associated taxa clustered together in the bootstrap test (1000 replicates) are shown next to the branches. The evolutionary distances were computed using the Poisson correction method expressed as the number of amino acid substitutions per site. Evolutionary analyses were conducted in MEGA7. *Right*, representative gene clusters of PlcP in human, animal and plant pathogenic *Burkholderia* spp. Numbers above each gene show the sequence identity to the corresponding gene in *B. cenocepacia* K56-2.

## Discussion

We report that under phosphate stress, *B. cenocepacia* produces three types of glyceroglycolipids (MGDG, KGADG, GADG) in a *plcP*/*agt-*dependent manner, suggesting that the PlcP phospholipase and the Agt glycosyltransferase play a critical role to drive membrane lipid remodeling in response to phosphate limitation. Our experiments also demonstrate that *plcP* is required for the intracellular survival of *B. cenocepacia* in mammalian macrophages and insect hemocytes. Using the *G. mellonella* infection model, we showed that during *B. cenocepacia* infection, PlcP transcriptional activity is rapidly expressed after bacterial engulfment (Figs. 5*A* and S5), suggesting that the *B. cenocepacia*-containing vacuole could be a low Pi environment. This notion agrees with the observation that Δ*plcP* cannot survive intracellularly (Fig. 3, *A* and *C*). The mutant displayed a higher initial infection rate in both the *G. mellonella* model (2 and 6 h post-infection) and macrophages (2 h post-infection) (Figs. 3*D* and 4*C*). Whether this is driven by an elevated rate of phagocytosis by host cells remains to be established. Indeed, the role of PlcP in *B. cenocepacia* pathogenesis could be due to several possibilities. Bacterial stress in the *B. cenocepacia*-containing vacuole may not only be due to scarce concentrations of important ions (*e.g.*, trace metals and phosphate) but also be attributed to increased production of oxidative reactive compounds. It has been shown previously that intracellular *B. cenocepacia* compromises phagosomal acidification and the assembly of the phagosomal NAPH oxidase in a Type VI secretion system (T6SS)-dependent manner by altering Rho family GTPases (33, 40). Increasing the intracellular Pi pool by releasing phosphate from phospholipids, PlcP may also influence the regulation of T6SS expression that is under the control of the global regulator AtsR (41). However, it is likely that PlcP's role in pathogenesis is multifaceted given that T6SS mutants are still able to survive intracellularly (42).

The association of *plcP* with a gene encoding a lipid glycosyltransferase (*agt*) implicates that PlcP plays a key role in the formation of novel membrane lipids whereby phosphate head groups are removed by PlcP-type enzymes and replaced by hydrophilic molecules such as carbohydrates. This pathway of membrane lipid remodeling is well-known in environmental microbes as an adaptation strategy to cope with phosphorus limitation (18, 19, 20, 26), and it remains to be seen whether environmental *Paraburkholderia* spp. use the same PlcP-Agt pathway for lipid remodeling. However, a role for this process in bacterial infection had not been previously described. The lipidomics data from bacterial cultures demonstrated that novel glycolipids appeared under Pi-depletion in a PlcP-dependent manner. Furthermore, promoter fusion assays demonstrated that *plcP* is actively transcribed during infection, arguing for a role of lipid remodeling during infection. Further experiments are required to unequivocally demonstrate that such alternative surrogate lipids appear in the bacterial membrane during infection. However, our data indicate that loss of PlcP/Agt-dependent membrane lipid remodeling could explain the reduced survival of the mutant strains, potentially due to their inability to manipulate the phagosome environment relative to the wild type strain, although the underline mechanisms remain unestablished. Alternatively, lipid remodeling may enable bacterial survival within the acidic lysosome, allowing the bacteria to persist long enough to secrete factors necessary for compartmental neutralization. Indeed, wild-type bacteria have been shown to arrest phagosomal acidification (34, 35, 36) or prevent being cleared by autophagy (43, 44) (Fig. 7). Other studies have also uncovered a role in virulence for bacterial membrane lipids. For example, the biosynthesis of sulfoglycolipids was directly associated with intracellular survival of *Mycobacterium tuberculosis* in human macrophages (45) and the control of phagosomal acidification (46). Moreover, a *B. cenocepacia* J2315 mutant lacking ornithine-containing aminolipids was attenuated in the *G*. *mellonella* infection model (47). Therefore, manipulating membrane lipids may be an important yet understudied mechanism for pathogenicity.

**Figure 7:**
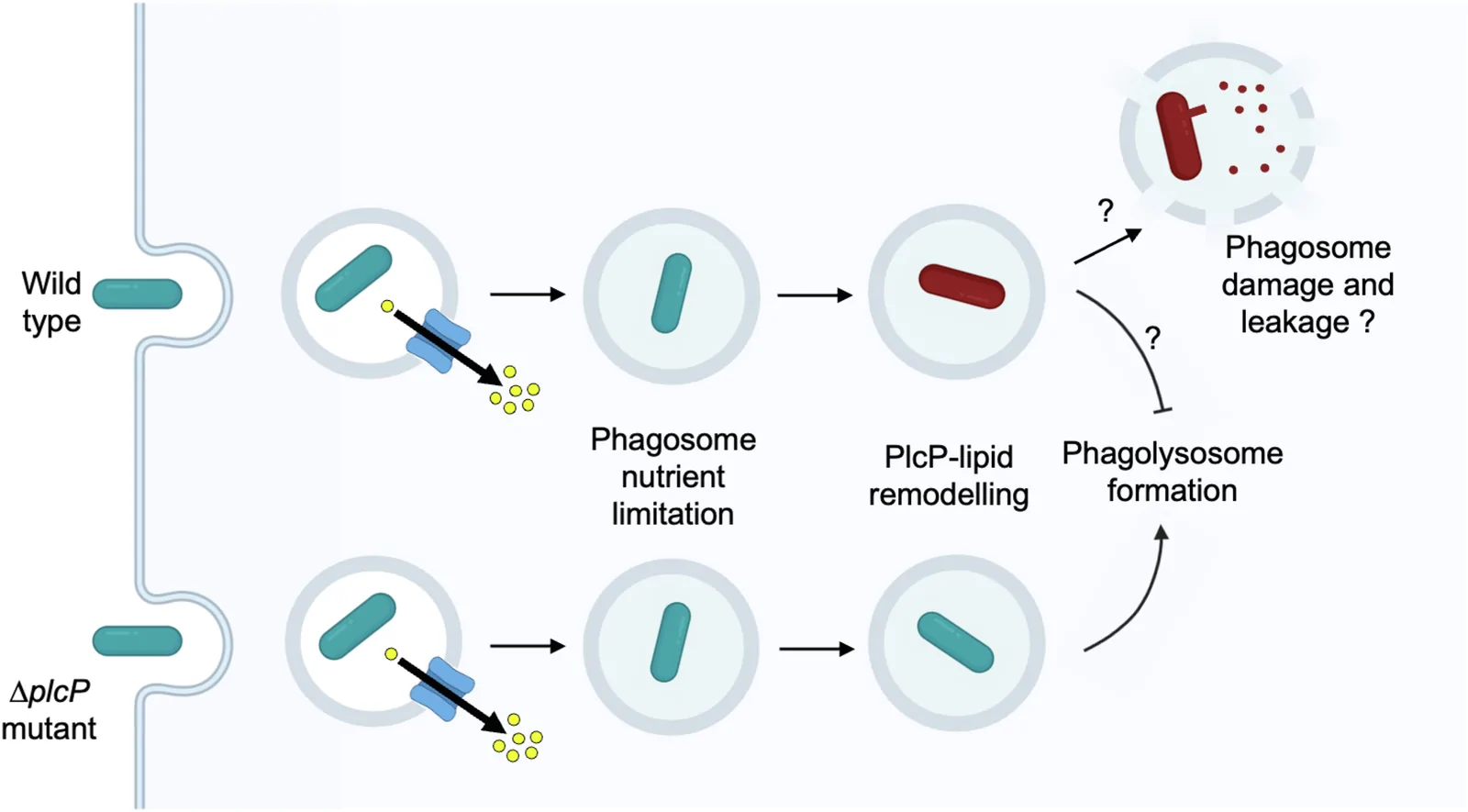
**Hypothetical model of the role of PlcP-mediated lipid remodeling during *B. cenocepacia* infection.** First, *B. cenocepacia* is taken up by immune cells by phagocytosis. The host likely restricts invading bacteria by limiting essential nutrients within the phagosome, such as phosphate. In response to nutrient limitation, the bacterium swiftly remodels its membrane and induces the formation of several glycolipids. The mechanisms by which membrane lipid remodeling enable the bacterium to better survive intracellularly remains unknown. Equally, it is unclear whether these lipid changes facilitate the secretion of the type VI effector TecA (*yellow dots*) or prevent phagosome damage and leakage. Additionally, lipid remodeling may allow the bacterium to subvert host autophagy pathways, further circumventing host-mediated restriction of the infection.

Given that the PlcP pathway is conserved in all pathogenic *Burkholderia* species, membrane remodeling could be a widely used strategy by this group of bacteria to survive in intracellular niches. Overall, our results underscore the role of phosphorus limitation as an overlooked element of nutritional immunity that may play a critical in the survival of intracellular bacteria (14, 15, 16, 17), which requires further exploration.

## Experimental procedures

### Growth of *B. cenocepacia* and alkaline phosphatase activity assays

A single colony of the respective *B. cenocepacia* strain was inoculated into 50 ml LB medium which was incubated overnight at 37 °C at 180 rpm. The optical density at 600 nm (OD_600_) was measured, and culture was centrifuged for 10 min at 4 °C at 3100*g* (4000 rpm, Eppendorf 5810R benchtop centrifuge, rotor A-4-81). Pelleted cells were resuspended in the MMA medium (21, 27) to 0.15 OD_600_. The high phosphate condition also included 1 mM Na_2_HPO_4_. At each timepoint after inoculation, alkaline phosphatase activity was measured with *p*-nitrophenol phosphate (*p*NPP, final concentration 1 mM), as an indicator of bacterial P stress. Quantification of *p*-nitrophenol (*p*NP) was followed at 405 nm in a 96-well plate in triplicate using a FLUOstar Omega plate reader (BMG Labtech).

### Cultivation of macrophages

Mouse macrophage RAW264.7 cell lines were obtained from ATCC and cultured with Dulbecco's Modified Eagle Medium (DMEM) supplemented with 10% FBS and L-glutamine at 37 °C, 5% CO_2_. Low-phosphate culture conditions during interaction with bacteria were achieved by using a modified Minimum Essential Medium (mMEM) with 10% FBS and L-glutamine containing 0.2 g/l CaCl_2_, 0.112 g/l MgSO_4_, 0.4 g/l KCl, 6.8 g/l NaCl, 1 g/l Glucose, 5.958 g/l HEPES, 2.2 g/l NaHCO_3_, 20 ml/l of MEM AA solution 50X (Gibco, Waltham, MA, USA), 10 ml/l of MEM Non-Essential Amino Acids Solution 100X (Gibco, Waltham, MA, USA) and 10 ml/l MEM Vitamin Solution 100X (Gibco, Waltham, MA, USA).

### Construction and complementation of the *plcP* and *agt* mutants in *Burkholderia cenocepacia* K56-2 and MH1K

*plcP* (BCAL2364) and *agt* (BCAL2365) genes were amplified by PCR and inserted into the suicide vector pGPI-SceI using Gibson assembly. Generation of *B. cenocepacia* deletion mutants was carried out as described previously (48). Complementation of the mutants was achieved by amplifying the gene together with the upstream intergenic region using PCR and Gibson assembly to fuse this region into the broad-host-range cloning vector pBBR1MCS-3 (49). The plasmids were then introduced into the corresponding Δ*plcP* or Δ*agt* mutant by conjugation. The mutants were confirmed by PCR amplification (Fig. S6) and subsequent Sanger DNA sequencing of the products.

### Labeling *B. cenocepacia* using fluorescent proteins and construction of a fluorescent reporter

To visualize *B. cenocepacia* wild type and Δ*plcP* mutant bacteria within phagocytic cells, plasmid pJT05 containing the fluorescent protein gene *mCherry* was introduced into the appropriate strains by conjugation. The number of bacteria expressing *plcP* during *G. mellonella* infections was quantified by using a reporter gene in which the native *plcP* promoter was fused with *mCherry.* The appropriate fragments were amplified by PCR and incorporated into the broad-host-range cloning vector pBBR1MCS-3 by Gibson assembly, resulting in *prPlcP::mCherry*. Controls containing only *mCherry* without a promoter (mCherry not expressed) and *mCherry* with the 200 bp upstream of *mCherry* in pJT05 (constitutively expressed) were constructed by the same method, and named *pr*∅*::mCherry* and *pr* constitutive*::mCherry*, respectively. These plasmids were introduced into *E. coli* WM3064 *via* electroporation and mobilized into WT *B. cenocepacia* K56-2 *via* conjugation. Briefly, overnight culture of WM3064 transformed with the desired plasmids was mated with overnight culture of *B. cenocepacia* in a 3:1 ratio on SOB plates containing 100 μg/ml of 2,6-diaminopimelic acid (DAP, Sigma-Aldrich) for 2 days at 37 °C. *B. cenocepacia* transconjugants were selected on DAP-free LB agar plates containing 150 μg/ml of tetracycline. To visualize bacteria not expressing mCherry, prior to the infection experiment *B. cenocepacia* cells were stained with 1X SybrGreen for 20 min.

### *B. cenocepacia* infection of macrophage cell lines

Prior to infection, WT *B. cenocepacia* and the *plcP* mutant strains were cultured in MMA broth until an OD_600_ of 0.6. To initiate infection of macrophages (RAW264.7), bacteria and macrophages were transferred to mMEM medium. Macrophage suspensions were inoculated into 24-well microplates at 5 × 10^5^ cells/ml containing a coverslip previously UV-sterilized and incubated overnight at 37 °C, 5% CO_2_ to initiate macrophages attachment. The bacteria (WT *B. cenocepacia* MH1K or the MH1K *plcP* mutant, both containing pJT05 constitutively expressing mCherry) were inoculated at a multiplicity of infection (MOI) of 2 (equivalent to 2 bacteria per macrophage cells). Low-speed centrifugation (5 min at 900*g*) was used to slowly move the bacteria down toward the adhered macrophages, and to initiate and synchronize physical interaction between bacteria and macrophages. The microplate was then incubated at 37 °C, 5% CO_2_ for 2 h. After 2 h post-infection (hpi), the cells were washed three times with mMEM to remove extracellular bacteria and a gentamicin protection assay (200 μg/ml) was performed for 2 h to kill any residual extracellular bacteria for the time points 6 h and 24 h. After 2 h, gentamicin was removed by three repeated washes with fresh mMEM. After 2, 6, 24 hpi, bacteria-macrophage interaction subsamples were stained with DAPI (5 μg/ml). Afterward, samples were directly fixed by adding formaldehyde (4% [vol/vol]) for 30 min. Finally, cells were mounted with a drop of Mowiol antifade before observation using confocal laser scanning microscopy (CLSM, Zeiss LSM 880, Göttingen, Germany).

To quantify bacterial load inside macrophages, macrophages suspended in mMEM were inoculated into 96-well microplates at 5 × 10^5^ cells/ml and incubated overnight at 37 °C, 5% CO_2_ to allow macrophage attachment. The bacteria (WT and Δ*plcP*) were inoculated at a MOI of 2 into 96-well microplates containing macrophages and low-speed centrifugation was used to initiate and synchronize physical interaction between bacteria and macrophages. The microplates were then incubated at 37 °C, 5% CO_2_. After 2 hpi, the cells were washed three times with mMEM to remove extracellular bacteria and incubated with gentamicin (200 μg/ml) for 2 h at 6 and 24 hpi. At each time point, macrophages were treated with Triton X-100 (0.05%, w/v) for 20 min at room temperature and mechanically lysed by mixing, using a syringe and 25-g needle (BD, Franklin Lakes, NJ, USA), as previously described (50). Bacteria were then enumerated by serial dilution on LB agar plates.

### Infection of *Galleria* hemocytes by *B. cenocepacia*

*G. mellonella* larvae (LiveFood) weighing 0.25 to 0.3 g and with no pigmentation were selected for experimentation within a 10-day period from their arrival (stored at 4 °C). Prior to infection, WT K56-2 *B. cenocepacia* and the K56-2 *plcP* or *agt* mutant strains were cultured in LB broth until an OD_600_ of 0.6. Bacterial cultures were resuspended in PBS 1X and a serial dilution was performed to reach a concentration of 20,000 bacterial cell/ml. A 500-μl Hamilton syringe equipped with an automatic dispenser and 27G needle was used to inject 10 μl of bacterial suspension into *G. mellonella via* the hindmost left proleg as previously described (51).

To assess the survival of the worms, 10 larvae per biological replicate were incubated in sterile Petri dishes after infection for 48 h at 37 °C and survival was monitored every 24 h by response to touch. One set of larvae was injected with sterile PBS 1X and one set of larvae was not injected, as controls to ensure the worms’ health.

Bacterial survival inside the larvae was estimated by isolating haemolymph from all 10 larvae in each biological replicate, culled after 48 hpi. Immediately after collection, haemolymph was serially diluted and 10 μl was plated onto LB containing 100 μg/ml gentamicin (Sigma-Aldrich) and 200 μg/ml ampicillin (Sigma-Aldrich) to kill any natural gut microbiota using the Miles and Misra method (52). Initial dose of bacteria was calculated by serially diluting the culture used for injection and plating 100 μl onto LB agar plates.

To observe the fate of the bacteria in the hemocytes of the insect, five larvae per sample were injected, alongside uninfected and PBS-injected controls. After 2, 6, 20 hpi, injected larvae were culled, and the hemolymph was extracted and resuspended in 1 ml of insect physiological saline (IPS), which consisted of: 150 mM NaCl, 5 mM KCl, 30 mM trisodium citrate, 10 mM EDTA, 100 mM Tris/HCl (pH 6.9) in sterile water; in a 24-well microplate containing a UV-sterilized glass coverslip. The haemocyte samples were then incubated for 1 h at 37 °C to initiate hemocytes attachment to coverslips. After incubation, the cells were washed three times with IPS to remove unattached hemocytes before staining with 70 nM Lysotracker Green (Thermofisher) and 5 μg/ml DAPI (Sigma-Aldrich) for 30 min at 30 °C, 60 rpm. Afterward, samples were directly fixed by adding formaldehyde (4% [vol/vol]) for 30 min. Finally, cells were mounted with a drop of Mowiol antifade before observation using CLSM.

To visualize bacteria inside the insect hemocytes by transmission electron microscopy (TEM), insect hemolymph was extracted after 20 hpi of the wild-type K56-2 infected larvae (n = 8), washed with IPS three times before fixing with 1 ml PBS containing 2.5% (w/v) glutaraldehyde. After three washes with PBS, the plates were then stored at 4 °C before sample preparation, which was completed by the Advanced Bioimaging RTP (The University of Warwick). Briefly, TEM samples were postfixed by immersion in 1% osmium tetroxide in water for 1 h, followed by staining with 2% uranyl acetate for 1 h. Samples were dehydrated using increasing acetone concentrations and directly embedded from the surface into Low Viscosity resin (Agar Scientific). Ultrathin (70 nm) sections were stained with uranyl acetate followed by lead citrate. Samples were imaged using a Jeol 2100Plus transmission electron microscope (Jeol) operating at 200 kV, equipped with a Gatan OneView IS detector (Gatan Ametek).

### Lipidomics analysis using LC-MS

A single colony of the respective *B. cenocepacia* strain was inoculated into the MMA medium to an OD_600_ of 0.15. Cells were grown for 8 h in the respective Pi-condition. Lipids were extracted using a modified Folch extraction method (53). A volume of cells equals to 1 ml of 0.5 OD_600_ was centrifuged for 15 min at 4 °C at 3100*g*. The supernatant was discarded and 50 μl lipid standard (10 μM), sphingosyl PE (SPE), was added giving a final concentration of 500 nM (Avanti Lipids). The pellet was then resuspended in 500 μl ice-cold methanol by vortexing. 300 μl ice-cold sterile water was added to the cells, followed by 1 ml ice-cold chloroform. The sample was vortexed for 30 s before being centrifuged for 15 min at 4 °C at 1739*g* (3000 rpm, Eppendorf 5810R benchtop centrifuge, rotor A-4-81). The lower phase containing the lipids was transferred to a fresh glass vial and the solvent was removed using a stream of nitrogen gas. Dried lipids were resuspended in 1 ml of acetonitrile:ammonium acetate (95:5, vol/vol). Samples were then analyzed by LC-MS using a Dionex Ultimate 3000-LC/MS (amaZon SL, Bruker). Lipids were separated based on the polarity of their head group on a BEH Amide XP column (Waters). The column was maintained at 30 °C with a flow rate of 150 μl/min. Each run consisted of a 10-min period of equilibration with 95% acetonitrile and 5% 10 mM ammonium acetate (pH 9.2). After this, 5 μl sample was injected into the system and was run on a 15 min gradient of 5 to 30% 10 mM ammonium acetate (pH 9.2). Each sample was analyzed in positive and negative ionization modes with auto fragmentation.

### Heterologous expression of *agt* in recombinant *E. coli*

To characterize the glycosyltransferase *agt*, it was chemically synthesized, and codon optimized by GenScript for overexpression in *E. coli*. Codon optimized *agt* gene was then cloned into the expression vector pET28a (Novagen) and chemically transformed into *E. coli* Lemo21(DE3) cells (New England Biolabs). Cells were then cultured overnight at 37 °C in the LB medium to an OD_600_ of ∼0.5 before induction with 0.5 mM IPTG. Cells were then harvested for lipidomics analyses as described earlier. Control experiments with the empty pET28a vector were also used.

### Statistics

For each experiment, at least three biological replicates were performed and ≥ 100 macrophages or haemocytes per replicates were counted. To test for statistically significant differences (*p* < 0.05) between two or more conditions, a *t* test or an ANOVA test was performed. These tests were performed by using SPSS 29.0 (IBM, Armonk, NY, USA).

## Data availability

Further information and requestion for resources and reagents, including plasmids and mutant strains generated in this study should be directed to Prof. Y Chen (y.chen.22@bham.ac.uk). Any additional information required to reanalyze the data reported in this paper is available from Prof Y Chen upon request.

## Supporting information

This article contains supporting information.

## Conflict of interest

The authors declare that they have no conflicts of interest with the contents of this article.
