# Supplementary material for: The phospholipase PlcP mediates membrane lipid remodeling required for the intracellular survival of Burkholderia cenocepacia
Source: J Biol Chem. 2026 Sep 23;302(11):113600. doi: 10.1016/j.jbc.2026.113600 (PMC13691135; doi:10.1016/j.jbc.2026.113600)
Supplement: Supplementary file 1 [file mmc1.docx]

**Supplementary Information**

**Supplementary Figure 1. Overexpression of *agt* in recombinant *E. coli* results in MGDG and GADG glycolipid production (blue lines).** Both lipids are absent in the empty plasmid control (black lines).

**Supplementary Figure 2. Combined heath index scores of wild type *Burkholderia cenocepacia* K56-2 and mutants infected insect larvae.** Each experiment was carried out with three biological replicates using 10 larvae per group. *** *p*<0.001

**Supplementary Figure 3.**  Partial plasmid loss during infection in *Galleria mellonella*. The *plcP* deletion mutant was complemented with the *plcP* gene, including its native promoter, amplified from the genomic DNA of *Burkholderia cenocepacia* K56-2 and cloned into the broad-host-range vector pBBR1MCS-3 carrying a tetracycline resistant cassette. The complemented strain was used to infect *G. mellonella*, and at 41 hours post infection, bacteria recovered from the haemolymph were plated on LB agar plates containing either ampicillin (to eliminate gut flora and select for *Burkholderia cenocepacia*) or tetracycline. A ~25-fold reduction in CFU counts was observed on tetracycline-containing plates compared with ampicillin plates, suggesting partial loss of the pBBR1MCS-*plcP* complementation plasmid during infection.

**Supplementary Figure 4. Transmission electron microscopy (TEM) image of wild type K56-2 infected insect haemocyte at 20-hour post infection (hpi).** Right-hand side panel shows zoomed in image of the haemocyte (red box of left-hand side panel). Bacterial cells are labelled BC.

**Supplementary Figure 5. Active transcription of the *B. cenocepacia* *plcP* gene in wild type cells during infection of *Galleria mellonella* using a promoter fusion assay.** Top panel, mCherry fused with the *plcP* promoter sequence. Middle panel, control plasmid of mCherry without the promoter. Bottom panel, control plasmid of constitutively expressed mCherry. Bacterial cells are stained with SybrGreen and the nucleus of haemocytes is stained with DAPI.

**Supplementary Figure 6 Construction and validation of *plcP* (top panel) and *agt* (bottom panel) deletion mutants in *Burkholderia cenocepacia*.** Two homologous regions located upstream and downstream of *plcP* were amplified with (forward/reverse primer, 5’-3’) gagctcgatatcgcatgcggtacctTTGAGCACCTTCGACTCC/ gtcatgcatgggcGCCTGACACAAC CCTACTTAC and ggttgtgtcaggcGCCCATGCATGACTCGCG/atccccgacggatcccaagcttcttCCTC GTTCATCACAACCTGC, respectively. These homologous regions were cloned into the suicide vector pGPI-SceI by Gibson assembly. The *plcP* deletion mutant was confirmed by PCR amplification using (forward/reverse primer, 5’-3’) GGCCCACGTACAGGAAGAT/ CCTCGTTCATCACAACCTGC and subsequent sequencing. To produce the *agt* knockout mutant, two homologous regions located upstream and downstream of *agt* were chemically synthesized by GenScript and cloned into the pUC57 vector under XbaI and SmalI sites, and the fragments were then subcloned into the suicide vector pGPI-SceI under the same restriction sites. The *agt* deletion mutant was confirmed by PCR using (forward/reverse primer, 5’-3’) CGATGGAAGGCGAACTGAAG/AGTTCAGCAGCTCGACGAT and subsequent Sanger DNA sequencing.
